# Emergency laparoscopic surgery for respiratory failure caused by perforated peptic ulcer and severe scoliosis: A case report

**DOI:** 10.1016/j.ijscr.2025.111020

**Published:** 2025-02-06

**Authors:** Kokichi Miyamoto, Makoto Matsumoto, Naoto Hori, Kenji Mizuno, Masato Kodera, Masahiro Oishi

**Affiliations:** Department of Surgery, Tottori City Hospital, 1-1 Matoba, Tottori-shi, Tottori-ken 6808511, Japan

**Keywords:** Severe scoliosis, Perforated peptic ulcer, Laparoscopic surgery

## Abstract

**Introduction:**

Laparoscopic surgery is increasingly performed for peptic ulcer perforation, but its application in patients with severe scoliosis remains controversial due to anatomical challenges and respiratory dysfunction. We report a case of a patient with severe scoliosis who developed respiratory failure from a perforated peptic ulcer and underwent emergency laparoscopic surgery.

**Presentation of case:**

A 51-year-old man was transferred to the hospital by ambulance for sudden abdominal pain. Localized tenderness was observed in the upper right quadrant. Upon admission, hypoxia developed with a PaO2 of 66.7 mmHg under oxygen therapy. Abdominal contrast-enhanced computed tomography (CT) imaging revealed a perforated peptic ulcer with free gas and ascites. Emergency laparoscopic surgery was performed safely using a customized port position.

**Discussion:**

Patients with scoliosis are prone to respiratory dysfunction, increasing their risk of life-threatening complications from peptic ulcer perforation. Early treatment is crucial in such cases. Meticulous perioperative management, appropriate port placement, and close communication with the anesthesiologist were key factors enabling emergency surgery in this high-risk patient, even if preoperative examinations could not be sufficiently conducted. Port insertion from the lower abdomen helped overcome anatomical limitations.

**Conclusion:**

Emergency laparoscopic surgery can be a viable option for patients with severe scoliosis and perforated peptic ulcers. Prompt decision-making, seamless perioperative management with the anesthesiologist, and tailored port placement to avoid intra-abdominal interference are essential for safe and effective procedures.

## Highlights

• Successful emergency laparoscopy performed in a patient with severe scoliosis.
• Customized port placement addressed anatomical constraints in scoliosis surgery.
• Close anesthetic collaboration ensured stable respiratory conditions.
• Early intervention prevented complications despite limited preoperative evaluations.
• Strategies for managing perforated peptic ulcers in high-risk scoliosis patients.

## 1 Introduction

Emergency surgery is the widely accepted standard treatment for perforated peptic ulcers [1]. Laparoscopic surgery has become increasingly common in recent years because it reduces mortality, complications, and postoperative pain [2]. However, its application in patients with severe scoliosis remains debatable due to anatomical challenges and respiratory dysfunction, which increases the risks associated with emergency surgery. We report a case of a patient with severe scoliosis who developed respiratory failure due to a perforated peptic ulcer and underwent emergency laparoscopic surgery. This work has been reported as being in line with the SCARE criteria [3].

## 2 Presentation of case

A 51-year-old man presented to the emergency department with sudden epigastric pain that began 3 h earlier. He also reported loss of appetite over the previous three days. His medical history included depression and abdominal surgery for appendicitis. While there was no history of surgery for the left kidney, computed tomography (CT) imaging revealed congenital left kidney deficiency. A *Helicobacter pylori* test was negative. Although he was prescribed four major tranquilizers for depression, he did not take any medications that could induce duodenal ulcers, such as non-steroidal anti-inflammatory drugs (NSAIDs). He smoked 20 cigarettes daily in his 20s but did not consume alcohol.

**Fig. 1:**
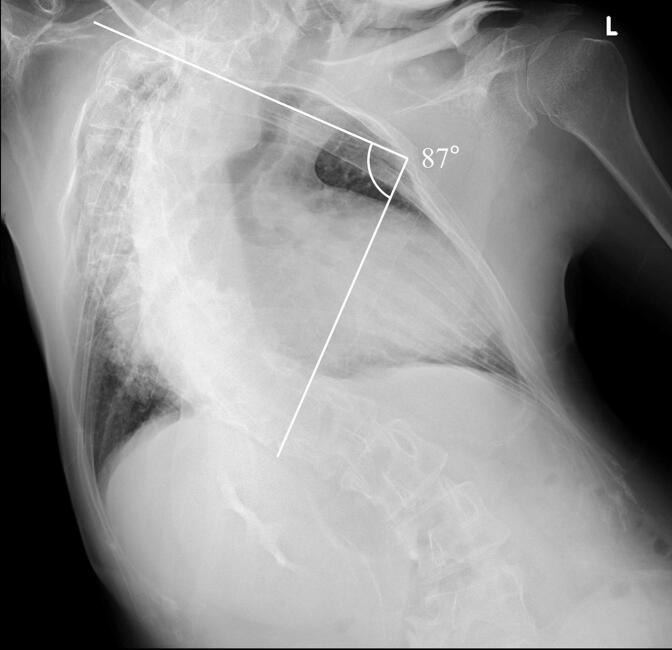
The patient had severe scoliosis. The curve of the spine was convex to the right at the level of the mid-thoracic esophagus and convex to the left at the level of the lower esophagus, with a Cobb angle of 87°.

On physical examination, his temperature was 37.0 °C, blood pressure was 107/86 mmHg, and heart rate was 120 beats per minute. Localized tenderness was observed in the right upper abdomen. Laboratory tests on admission are summarized in Table 1. Despite oxygen administration, hypoxemia persisted with a partial pressure of oxygen (PaO2) of 66.7 mmHg, raising suspicion of respiratory failure. The patient also had severe scoliosis with a thoracic convexity to the right at the mid-thoracic esophagus and a lumbar convexity to the left at the lower esophagus, with a Cobb angle of 87° (Fig. 1.)

**Table 1:** Laboratory data on admission.

WBC	9400	/μL	AST	235	U/L
RBC	554 × 10^4^	/μL	ALT	766	U/L
Hgb	17.6	g/dL	γGT	40	U/L
Hct	55.8	%	LDH	444	U/L
PLT	31.3 × 10^4^	/mm^3^	T-Bil	1.6	mg/dL
TP	5.5	g/dL			
Alb	3.2	g/dL	PT	70.8	%
Cre	0.71	mg/dL	APTT	30.6	s
BUN	30.0	mg/dL			
Na	135	mmol/L	pH	7.373	
K	4.0	mmol/L	PaO2	66.7	mmHg
Cl	89	mmol/L	PaCO2	57.6	mmHg
GLU	110	mg/dL	HCO^3−^	29.5	mmol/L
CRP	5.41	mg/dL

**Fig. 2:**
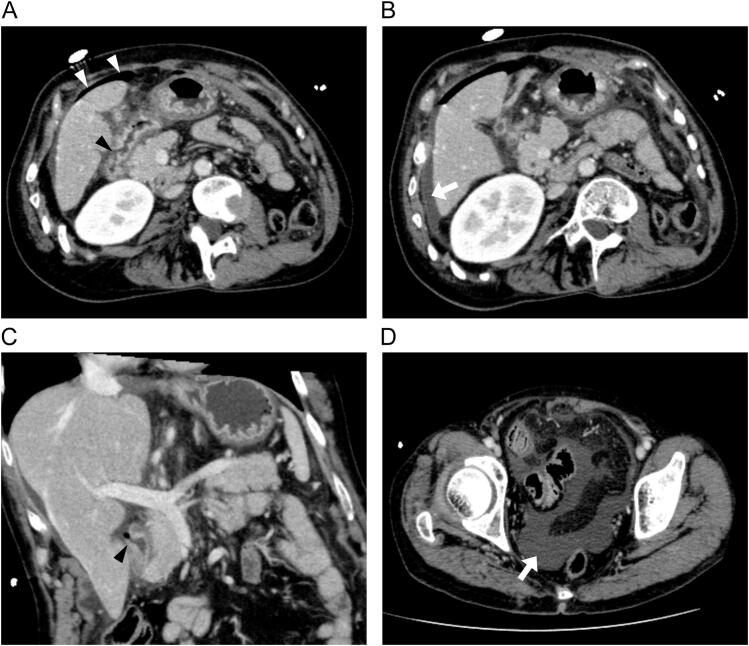
Abdominal contrast-enhanced CT revealed a discontinuity in the duodenal wall (black arrowhead), free gas on the surface of the liver (white arrowhead), and a small amount of ascites (white arrow) (panels A, B, C). A moderate amount of ascites (white arrow) was also observed in the Douglas fossa (panel D).

Abdominal contrast-enhanced CT revealed discontinuity in the duodenal wall, free gas over the liver surface, and a small amount of ascitic fluid (Fig. 2A–C), consistent with duodenal bulb perforation. Additionally, moderate ascites was present in the Douglas fossa (Fig. 2D). Based on these findings, emergency laparoscopic surgery was performed for a confirmed diagnosis of duodenal ulcer perforation.

**Fig. 3:**
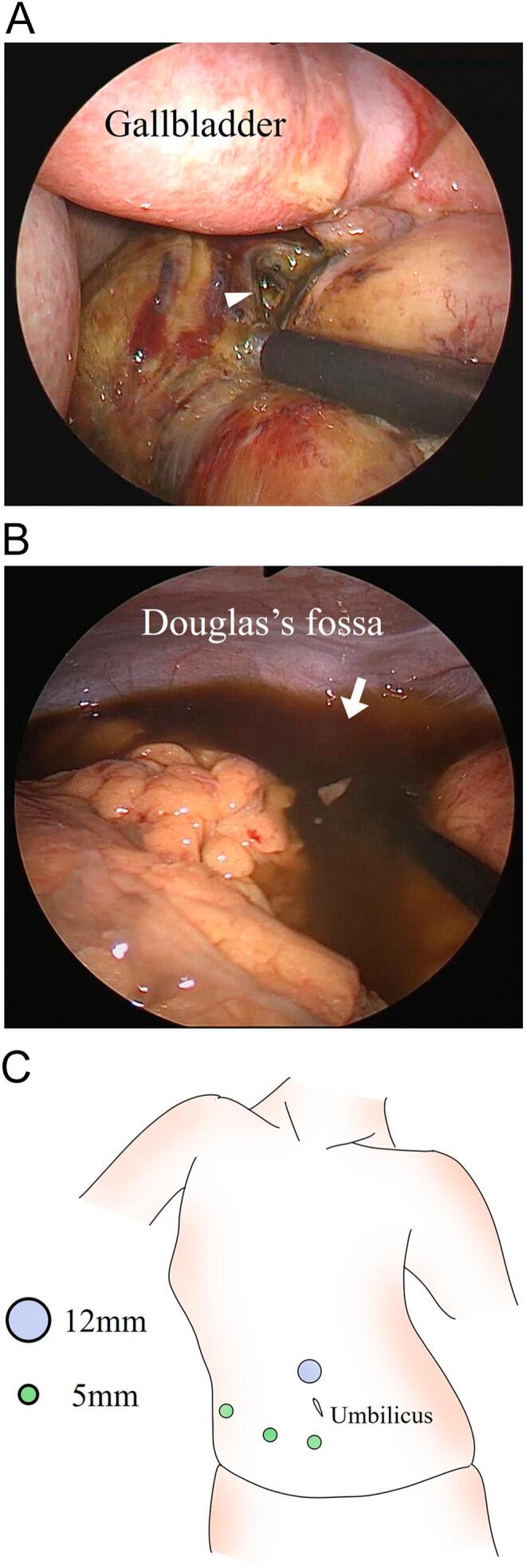
Laparoscopy revealed that the anterior wall of the duodenum was perforated by approximately 10 mm (white arrowhead) and that biliary ascites had spread throughout the abdomen (white arrow) (panels A, B). The schema of port placement on laparoscopic surgery (panel C).

A 5-mm diameter, 30°-angled laparoscope showed a 10-mm perforation in the anterior duodenal wall, with biliary ascites throughout the abdomen (Fig. 3A, B). Three additional ports were placed (Fig. 3C), and laparoscopic primary suturing, omental patch repair, and peritoneal lavage with 10 L of saline were performed. Multichannel drains were inserted into the Morrison's and Douglas fossa. Postoperative management included treatment for atelectasis caused by sputum accumulation, enabling extubation on postoperative day four. The drainage tubes were removed on postoperative days three and five, without any intra-abdominal infectious complications. Oral intake resumed on postoperative day seven. Other than immediate postoperative respiratory management after surgery, the patient experienced no complications and was discharged on postoperative day 22.

## 3 Discussion

Here, we present a case of severe scoliosis in a patient who developed respiratory failure due to a perforated peptic ulcer and safely underwent emergency laparoscopic surgery. Key factors contributing to this successful outcome included prompt decision-making, meticulous perioperative management, and strategic port placement. These factors enabled us to perform emergency surgery despite the patient's unstable general condition and the limitations of insufficient preoperative examinations.

The Japanese Society of Gastroenterology (JSGE) recommends emergency surgery for peptic ulcer perforation in patients over 70 years, with onset exceeding more than 24 h, serious comorbidities, unstable hemodynamics, peritonitis symptoms, or massive gastric contents or ascites [4]. In this case, the patient was rapidly diagnosed after symptom onset, and imaging findings did not show extensive features warranting emergency surgery. While the patient had localized abdominal pain in the right quadrant, overt signs of peritonitis were minimal. However, scoliosis posed an underlying risk for respiratory compromise, as evidenced by hypoxia that developed shortly after symptom onset. Early surgical intervention was, therefore, prioritized, and the procedure was initiated approximately 2 h after the patient arrived at the hospital. Laparoscopy revealed peritonitis with bilious ascites extending into the Douglas fossa, underscoring the importance of prompt surgical intervention in achieving favorable outcomes. To the best of our knowledge, there have been no reports of scoliosis patients who have suffered from perforated peptic ulcers, so it remains controversial whether they all require emergency surgery. However, therapeutic decisions should be tailored to the patient's clinical background, even when laboratory test results or imaging findings are not pronounced.

Emergency surgery for scoliosis patients necessitates more meticulous perioperative management compared to elective procedures. Limited patient information was efficiently shared with the anesthesiologist, allowing surgery to begin within 2 h of hospital arrival. Delayed surgery has been associated with increased mortality risk [1], highlighting the importance of timely intervention. Laparoscopic surgery was chosen for its advantages, including a magnified view and reduced postoperative pain. The operation was conducted under 10 mmHg pneumoperitoneum pressure, with continuous communication between the surgical and anesthetic teams to ensure stable intraoperative respiratory conditions. While some studies recommend a pneumoperitoneal pressure of 6 mmHg for laparoscopic surgery performed on scoliosis patients to account for respiratory dysfunction, others, including this case, have demonstrated the feasibility of higher pressures [5,6]. Although laparoscopic surgery was performed under stable respiratory conditions in this case, it is vital to consider reducing insufflation pressure or converting to laparotomy in cases of instability.

Postoperative management involved general anesthesia with intubation due to the unassessed baseline respiratory conditions. Hypoventilation, likely due to scoliosis and sputum retention, resulted in hypercapnia. However, treatment for atelectasis and adequate recovery from peritonitis prevented the need for tracheotomy, reintubation, or respiratory failure after extubation. Close collaboration with anesthesiologists and comprehensive perioperative management, not only during surgery but also after surgery, were essential factors for successful outcomes.

Proper trocar placement is crucial for efficient laparoscopic procedures. The first port was a 12-mm trocar placed above the umbilicus, similar to those used in laparoscopic cholecystectomy or distal gastrectomy. Intra-abdominal exploration revealed a restricted working space, especially in the right upper abdomen due to scoliosis. To address this, three additional 5-mm ports were placed in the lower abdomen, with the initial port serving as the primary manipulation site. These placements minimized intra-abdominal interference, enabling the successful completion of laparoscopic primary suturing, omental patch repair, and peritoneal lavage within 120 min, a duration comparable to previously reported operative times for laparoscopic surgeries for perforated peptic ulcers [7].

Although all ports in this case were inserted intraperitoneally, some reports suggest the feasibility of intercostal ports to overcome limitations in laparoscopic surgeries for patients with turtle-back deformities [8]. Such adaptations may enhance the safety and efficiency of laparoscopic surgery in scoliosis patients when necessary.

## 4 Conclusion

Emergency laparoscopic surgery may be a viable option for patients with severe scoliosis and perforated peptic ulcers. Safe and effective procedures require prompt decision-making regarding treatment strategies, seamless perioperative collaboration with the anesthesiologist, and appropriate port placement to minimize intra-abdominal interference.

## Consent

Written informed consent was obtained from the patient for publication of this case report and any accompanying images. A copy of the written consent is available for review by Editor-in-Chief of this journal on request.

## Ethical approval

Tottori City Hospital does not require ethical approval for individual case reports.

## Guarantor

Dr. Kokichi Miyamoto accepts full responsibility for the work and conduct of this study. He had access to the data and controlled the decision to publish the findings.

## Sources of funding

We received no financial support for the publication of this article.

## Research registration

None.

## Author contribution

The idea and content of the manuscript were conceived by KM.

All clinical assessments and the surgical procedure were performed by KM and MM.

Data collection and interpretation, including radiographic analysis, were conducted by KM and MM. The initial draft of the manuscript was written by KM.

KM, MM, and MO reviewed, edited, and approved the final version of the manuscript.

## Declaration of competing interest

We have nothing to disclose.
